# SNAI2 promotes the development of ovarian cancer through regulating ferroptosis

**DOI:** 10.1080/21655979.2021.2024319

**Published:** 2022-02-27

**Authors:** Yunfeng Jin, Li Chen, Li Li, Guoqin Huang, Hua Huang, Chunhui Tang

**Affiliations:** a Department of Gynecology, Obstetrics and Gynecology Hospital, Fudan University, Shanghai, China; b Department of Obstetrics and Gynecology, Affiliated Hospital of Nantong University, Nantong, China; c Department of Obstetrics and Gynecology, Affiliated Maternity and Child Health Care Hospital of Nantong University, Nantong, China

**Keywords:** SNAI2, ovarian cancer, erastin, ferroptosis, SCL7A11

## Abstract

This study aims to explore the regulatory mechanism of SNAI2 in ovarian cancer, and to uncover its correlation with ferroptosis. A human normal ovarian cell line IOSE-80 and four ovarian cancer cell lines (SKOV3, A2780 and CAOV3) were applied to detect SNAI2 and ferrptosis level, and an elevated SNAI2 expression and the occurrence of ferroptosis were observed in ovarian cancer cells, especially in SKOV3 cells. Then, results from a series of cellular behaviors experiments revealed that SNAI2 knockdown greatly suppressed cell viability, migration, invasion, and promoted cell apoptosis, as well as promoting the occurrence of ferroptosis in SKOV3 cells. The effects of SNAI2 knockdown on SKOV3 cells were similar to erastin, an inducer of ferroptosis. Subsequently, SNAI2 was verified to directly bind to the promoter of SLC7A11 by luciferase reporter assay and chromatin immunoprecipitation (ChIP) assay. Furthermore, mice were subcutaneously injected with SKOV3 cells to induce tumor formation. Erastin exhibited an anti-tumor effect on mice suffering from ovarian cancer, which was partly weakened by SNAI2 overexpression. In conclusion, this study disclosed that SNAI2 knockdown or erastin exhibited an anti-tumor activity in ovarian cancer by promoting ferroptosis, shedding new insights of the regulatory mechanism of SNAI2-mediated ferroptosis in ovarian cancer.

## Graphical abstract

**Figure:**
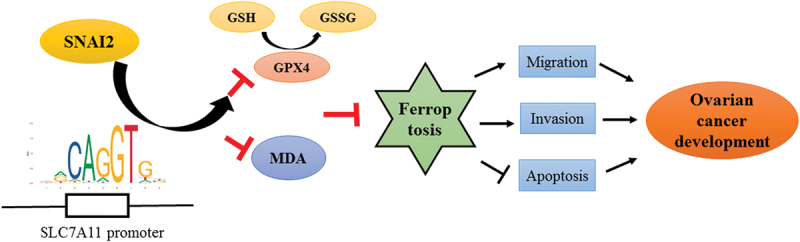

## Introduction

Ovarian cancer is the deadliest malignant tumor among female reproductive carcinoma worldwide, including China [1]. The vagueness of symptoms resulted in delayed diagnosis and higher tumor stages for the majority of women. Currently, the mainstream therapy for ovarian cancer includes surgery followed by platinum-based chemotherapy. While the patients initially respond favorably to this therapy, the majority of them relapse with a recurrent disease, leading to incurable disease with limited treatment options [2]. Thus, the five-year survival rate of ovarian cancer is still unsatisfactory [3]. Therefore, it is important to identify key predictive biomarkers and precise mechanism of action to provide effective therapeutic strategies of ovarian cancer treatment and to pro-long the survival time of ovarian cancer patients.

Ferroptosis is a novel form of iron-dependent programmed cell death characterized by iron-caused accumulation of lipid peroxide [4]. Recent evidence reveals that ferroptosis regulates tumor genesis, progression and metastasis, and targeting ferroptosis may be a feasible strategy for cancer therapy, including ovarian cancer [5–7]. A novel ferroptosis-related gene signature has been observed to be associated with the prognosis in patients with ovarian cancer, identifying the critical role of ferroptosis-related hub genes during ovarian cancer development [8]. Solute carrier family 7 member 11 (SLC7A11) is a cystine/glutamic acid Xc-transport carrier responsible for the specific transport of cystine and glutamine, and acts as the rate-limiting precursor for glutathione (GSH) biosynthesis [9]. GSH is a lipid peroxide scavenger and serves as a cofactor for glutathione peroxidase (GPX4) to reduce the accumulation of lipid-based reactive oxygen species (ROS), thereby protecting cells from ferroptosis [10–12].

SNAI2 (also known as Slug) encodes a zinc-finger protein of the SNAI family of transcription factors [13]. Recent evidence reveals a broad effect of SNAI2 on cancer progression, including inducing tumor initiation cell, promoting cell invasion and metastasis [14,15]. Elevated expression of SNAI2 has been observed in multiple cancers, which was also positively linked to the shorter survival time and the increased risks of recurrence and metastasis for patients [16–18]. In terms of ovarian cancer, SNAI2 was frequently activated in the tumor stroma, and has potential to promote lymphovascular spread of ovarian cancer [19,20]. In addition, SNAI2-3ʹ untranslated regions (UTR) cloud promote the invasion of ovarian cancer cells, further emphasizing the tumor-promoting action of SNAI2 in ovarian cancer. However, the potential mechanism underlying the regulation of SNAI2 in ovarian cancer is limited.

Interestingly, a predicted binding site between the promoter of SLC7A11/GPX4 and SNAI2 was found from JASPAR bioinformatics website (http://jaspar.genereg.net/), indicating a potentially regulatory effect of SNAI2 on SLC7A11 and GPX4. Considering the importance of SLC7A11 and GPX4 in ferroptosis, we speculated that SNAI2 might be involved in the regulation of ferroptosis, which accounted for its role in ovarian cancer. The goal of this study is to clarify the connection among SNAI2, ferroptosis and ovarian cancer, and to uncover the potential regulatory mechanism.

## Methods

### Cell culture

The human normal ovarian cell line IOSE-80 was purchased from Yaji Biological (Shanghai, China), and three ovarian cancer cell lines SKOV3, A2780 and CAOV3 were obtained from BeNa Culture Collection (Beijing, China). A2780 and CAOV3 cells were cultured in Dulbecco’s Modified Eagle’s Medium (DMEM; Hyclone, USA) supplemented with 10% fetal bovine serum (FBS; Gibco, USA). IOSE-80 and SKOV3 cells were cultured in Roswell Park Memorial Institute (RPMI)-1640 medium (Thermo Fisher Scientific, Inc.) containing 10% FBS. All cells were incubated at 37°C in an incubator with an atmosphere of 5% CO_2_.

### Cell Counting Kit-8 assay

Cell proliferation ability was determined using Cell Counting Kit-8 (CCK-8) assay. Briefly, SKOV3 cells were seeded into 96-well plates (5 × 10^3^ cells/well). After incubation for 24 h, 48 h, and 72 h, respectively, 10 μl of CCK-8 agent (Beyotime Institute of Biotechnology, China) was added to each well, and cells were incubated at 37°C in an incubator with an atmosphere of 5% CO_2_ for another 2 h. Finally, the absorbance at 450 nm of each well was measured using a microplate reader (Bio-Rad Laboratories, Inc., Hercules, CA, USA).

### Transwell assay

Cell invasion ability was detected using Transwell assay. In brief, cells were resuspended in serum-free medium and seeded on Matrigel (BD Biosciences) precoated inserts of a 24-well plates, while regular complete medium containing 10% FBS was added to the wells. 24 h later, cells were fixed with 4% paraformaldehyde for 15 min and stained with 4% crystal violet for 25 min at room temperature. The invasive cells were observed and counted using a light microscope (DM1000; Leica Microsystems GmbH, Wetzlar, Germany).

### Wound-healing assay

Cell migration ability was detected using wound-healing assay. Briefly, SKOV3 cells were seeded into 6-well plates (1 × 10^4^ cells/well). When reaching 100% confluence, a scratch was generated using a 20-μl pipette tube. The debris was wiped out by washing with phosphate buffer saline (PBS), and the serum-free medium was used for the further incubation. At 0 h and 24 h, the images were captured using a light microscope and the migration rate was assessed.

### The detection of biochemical factors

The content of malondialdehyde (MDA) in cell lysates was determined by lipid peroxidation assay kit (Beyotime Institute of Biotechnology, China) according to the manufacturer’s instructions. The level of the reduced (GSH) and oxidized (GSSG) forms of glutathione was measured using a GSSG/GSH quantification kit (elabscience, China) following the manufacturer’s instructions.

### Iron assay

Intracellular Fe^2+^ levels were determined using an Iron Colorimetric Assay kit (BioVision, Inc., California, USA) and the fluorescent probe Phen green SK (Invitrogen, Carlsbad, CA, USA) strictly in line with the manufacturers’ instructions, respectively. For the colorimetric method, the absorbance at the wavelength of 593 nm was detected with a microplate reader. For the fluorescent probe method, the images were captured by a fluorescence microscope (Olympus, Tokyo, Japan).

### Cell transfection

Short hairpin targeting SNAI2 (shRNA-SNAI2) and the negative vector were obtained from Shanghai GenePharma co., ltd. 3 × 10^5^ SKOV-3 cells were seeded in a 3.5-cm dish at 37°C in an incubator with an atmosphere of 5% CO_2_. When reaching 80% confluence, the vectors and Lipofectamine 3000 reagent (Thermo Fisher Scientific, Inc.) were diluted with 125 μl Opti-MEM medium for 5 min, respectively, and then were mixed for 20 min, followed by incubation with SKOV-3 cells for transfection. The dose of shRNA-SNAI2/vector was 500 ng/μl. 48 h post transfection, the transfected cells were harvested for subsequent experiments.

### Western blot

Total protein was extracted using radioimmunoprecipitation assay (RIPA) lysis buffer (Beyotime Institute of Biotechnology, China), followed by determining the protein concentration using a Protein Assay Kit (Pierce). The same amount of protein (30 μg/lane) was separated using 12% sodium dodecyl sulfate polyacrylamide gel electrophoresis (SDS-PAGE), and then transferred onto polyvinylidene fluoride (PVDF) membranes (Millipore). After blocking with 5% fat-free dried milk at room temperature for 1 h, the membranes were incubated at 4°C overnight with primary antibodies against SNAI2 (dilution 1:1,000, cat. no. ab180714, Abcam), SLC7A11 (dilution 1:1,000, cat. no. ab37185, Abcam), GPX4 (dilution 1:1,000, cat. no. ab125066, Abcam), Bcl-2 (dilution 1:2,000, cat. no. ab182858, Abcam), Bax (dilution 1:1,000, cat. no. ab32503, Abcam), cleaved caspase3 (dilution 1:1,000, cat. no. orb106556, Biorbyt), ACSL4 (dilution 1:10,000, cat. no. ab155282, Abcam), NCOA4 (dilution 1:1,000, cat. no. orb540576, Biorbyt), TFR1 (dilution 1:1,000, cat. no. ab214039, Abcam), DMT1 (dilution 1:800, cat. no. 20,507-1-AP, Proteintech) and GAPDH (dilution 1:2,500, cat. no. ab9485, Abcam). On the following day, the membranes were incubated with horseradish peroxidase-conjugated goat anti-rabbit IgG secondary antibody (dilution 1:2,000, cat. no. ab6721, Abcam) at room temperature for 2 h. The signals were developed using an enhanced chemiluminescence detection kit (Sigma-Aldrich; Merck KGaA, Darmstadt, Germany) and imaged by the Tanon 5200 chemiluminescence imaging system (BioTanon, China), followed by quantification using ImageJ software (version 1.50; National Institutes of Health).

### Quantitative real-time PCR (qRT-PCR)

Total RNA was extracted from cells using Trizol (Invitrogen) in guide of the manufacturer’s protocol. RNAs were reverse-transcribed into cDNA using Reverse Transcription kit (Promega). qRT-PCR was carried out using SYBR Green PCR mix (DSBIO) in ABI PRISM 7500 Sequence Detection System (Applied Biosystems). mRNA levels were calculated using Comparative CT method and normalized to β-actin. Primers used in this study was shown as follows: SNAI2 (forward: 5ʹ- CTCTCTCCTCTTTCCGGATACT-3ʹ; reverse: 5ʹ- GCTTGGACTGTAGTCTTTCCTC-3ʹ), SLC7A11 (forward: 5ʹ- GGTTGCCCTTTCCCTCTATTC-3ʹ; reverse: 5ʹ-CCTGGGTTTCTTGTCCCATATAA-3ʹ), GPX4 (forward: 5ʹ- GTGAGGCAAGACCGAAGTAAA-3ʹ; reverse: 5ʹ- GAACTGGTTACACGGGAAGG-3ʹ) and β-actin (forward: 5ʹ- CGGGAAATCGTGCGTGAC-3ʹ; reverse: 5ʹ-CAGGAAGGAAGGCTGGAAG-3ʹ).

### Luciferase reporter assay

The binding site between SLC7A11 promoter and SNAI2 was predicted by JASPAR bioinformatics website (http://jaspar.genereg.net/). The wild-type (WT) and mutant (MUT) sequences of SLC7A11 promoter were synthesized and inserted downstream of pGL3-control luciferase reporter vectors (Promega corporation) to generate SLC7A11-WT and SLC7A11-MUT. SKOV3 cells were co-transfected with SLC7A11-WT/SLC7A11-MUT and pcDNA3.1 or pcDNA3.1-SNAI2 using Lipofectamine 3000 (Thermo Fisher Scientific, Inc.) according to the manufacturer’s instructions. 48 h post transfection, the luciferase activities were measured using the dual-luciferase reporter assay kit (Promega corporation).

### Chromatin immunoprecipitation (ChIP) assay

SKOV3 cells were cross-linked with 1% formaldehyde at room temperature for 10 min and quenched by glycine. Subsequently, the cell lysates were sonicated to generate chromatin fragments, followed by immunoprecipitation with anti-SNAI2 or the negative control IgG at 4 °C overnight. The precipitated DNA was purified and then detected by qRT-PCR [21].

### In vivo experiments

All procedures and protocols involving mice were approved by the Institutional Animal Care and Use Committee of Affiliated Hospital of Nantong University. Total 24 nude female BALB/cA-nu mice (6-weeks-old) were obtained from Shanghai SLAC Laboratory Animal Co., Ltd. (Shanghai, China) and housed in a constant environment (12 h light/dark cycle; temperature, 22 ± 2°C; humidity, 50‑60%) with free access to water and food. After acclimation for 1 week, all mice were randomly assigned into four groups: i) Control: mice received subcutaneous injection into the flanks of SKOV3 cells (2 × 10^6^) to induce tumor formation; ii) Erastin: mice received erastin treatment (30 mg/kg intraperitoneally, twice every other day) [22]; iii) Erastin+Blank: mice received erastin treatment and injection of SKOV3 cells transfected with blank lentiviral vector; iv) Erastin+SNAI2: mice received erastin treatment and injection of SKOV3 cells transfected with lentiviral vector of SNAI2 overexpression. The mice weight and tumor volume were monitored every 3 days. Tumor volume based on caliper measurements was calculated as follows: volume = (length × width^2^)/2. Three weeks after beginning treatment, the mice were sacrificed and the tumors were harvested for subsequent analysis.

### Immunohistochemical (IHC) assay

The tumors were fixed in 4% paraformaldehyde, embedded in paraffin, and cut into sections with 4-μm thickness. After dewaxing in xylene and rehydration in descending concentrations of ethanol, the sections were heated in 0.01 M citric buffer (pH 6.0) for 15 min with autoclaving, followed by cooling to room temperature. Subsequently, the sections were treated with 1% H_2_O_2_ in PBS for 5 min to block endogenous peroxidase, and incubated for 1 h in PBS containing 5% normal goat serum. After washing, sections were incubated with the primary antibody against SNAI2 (dilution 1: 100, cat. no. ab180714, Abcam) at 4°C overnight, and then with horseradish peroxidase-conjugated secondary antibody (dilution 1:1,000, cat. no. ab6721, Abcam) for 30 min at room temperature, followed by diaminobenzidine (DAB) staining (ZSGB-BIO, China). Finally, the sections were counterstained with hematoxylin, and the images were captured under an Olympus microscope (magnification × 200; BX43, Olympus, Japan).

### Statistical analysis

The data were analyzed using GraphPad Prism version 8.0 and expressed as the mean value ± standard deviation. The student’s t-test was applied to compare the differences between two groups, while comparisons among more than two groups were determined with one-way ANOVA assay followed by Tukey’s post hoc test. P value less than 0.05 was considered to indicate statistically significant.

## Results

### SNAI2 and ferroptosis were correlated with ovarian cancer

**Figure 1.**
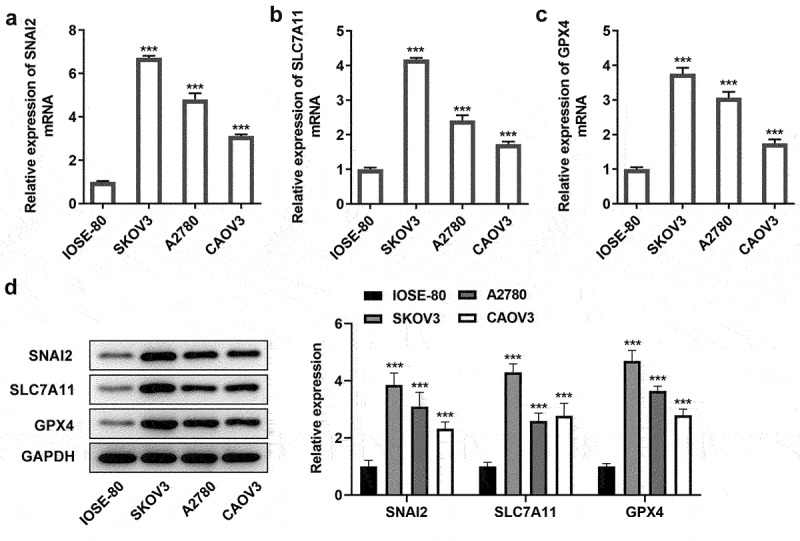
The expression level of SNAI2, SCL7A11 and GPX4 in ovarian cancer. The expression level of SNAI2, SLC7A11 and GPX4 in human normal ovarian cell line IOSE-80 and multiple ovarian cancer cells (SKOV3, A2780 and CAOV3) were detected using (a-c) qRT-PCR and (d) Western blot, respectively. ***p < 0.001 vs IOSE-80.

**Figure 2.**
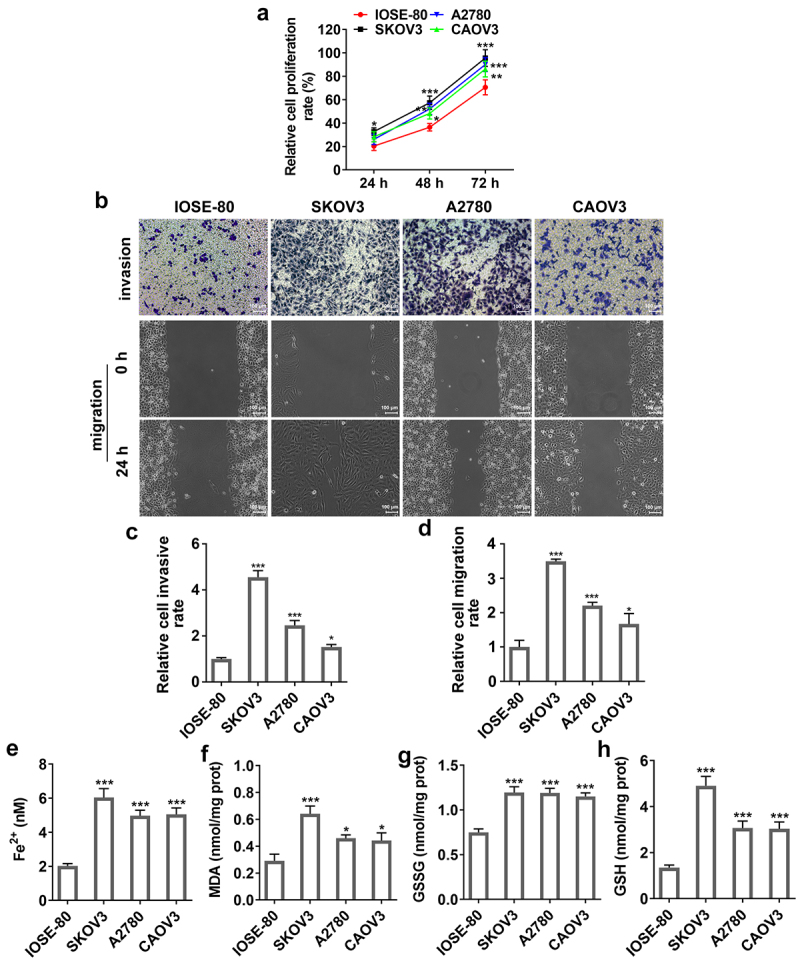
The migration, invasion and ferroptosis in ovarian cancer. The human normal ovarian cell line IOSE-80 and multiple ovarian cancer cells (SKOV3, A2780 and CAOV3) were used for analysis. (a) Cell proliferation was detected using CCK-8 assay. (b-d) The invasion ability and migration ability of different cell lines were measured by Transwell assay and wound-healing assay, respectively. The content of (e) Fe^2+^, (f) MDA, (g) GSSG and (h) GSH were detected using their corresponding commercial kits. *p < 0.05, **p < 0.01, and ***p < 0.001 vs IOSE-80.

Firstly, to identify the connection among SNAI2, ferroptosis and ovarian cancer, the expression level of SNAI2, SLC7A11 and GPX4 in human normal ovarian cell line IOSE-80 and multiple ovarian cancer cells were detected. The results from qRT-PCR and Western blot assays showed that both the mRNA level and protein expression of SNAI2, SLC7A11 and GPX4 in SKOV3, A2780 and CAOV3 cells were higher than that in IOSE-80 (Figure 1a-d), suggesting that SNAI2, SLC7A11 and GPX4 were upregulated in ovarian cancer. Then, a series of cellular behavior experiments revealed that the cell proliferation, migration and invasion abilities of ovarian cancer cells (KOV3, A2780 and CAOV3 cells) were higher than those in IOSE-80 cells (Figure 2a-d). In addition, the levels of MDA and GSSG were greatly upregulated, while the levels of Fe^2+^ and GSH in ovarian cancer cells, compared to IOSE-80 cells (Figure 2e-h), suggesting ferroptosis was activated in ovarian cancer, considering with the high level of SLC7A11 and GPX4 in ovarian cancer described above. In particular, due to the most obvious features of aberrant SNAI2 expression, ferroptosis occurrence and cellular behaviors in SKOV3 cells, SKOV3 cells were applied for the further experiments.

SNAI2 knockdown suppressed cell migration, invasion and induced apoptosis by inducing ferroptosis

**Figure 3.**
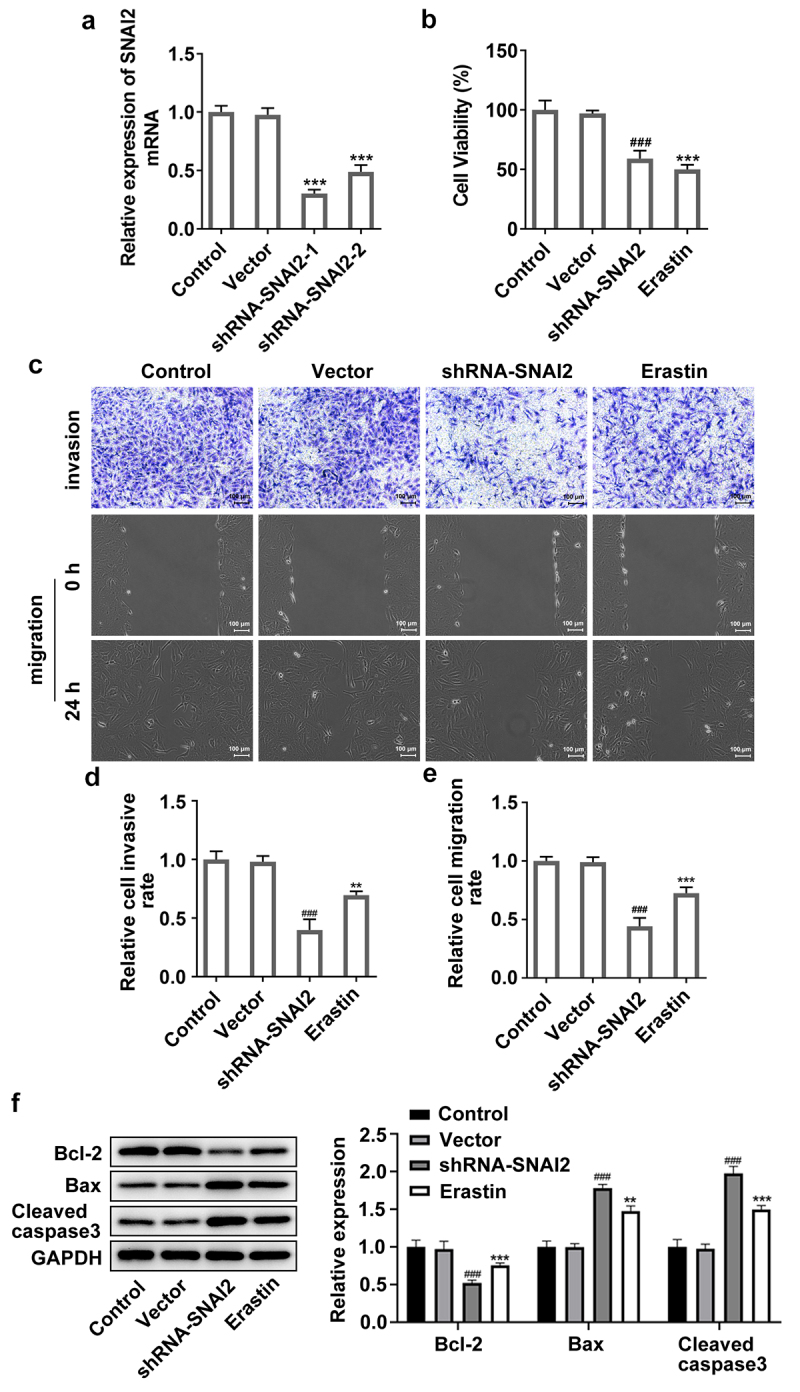
The effect of SNAI2 knockdown on cell migration, invasion and apoptosis in SKOV3 cells. (a) SKOV3 cells were transfected with shRNA-SNAI2-1/2 and its control (vector), and the mRNA level was detected using qRT-PCR 48 h after transfection. ***p < 0.001 vs vector. SKOV3 cells were transfected with shRNA-SNAI2 or vector, or received erastin treatment (30 μM). The (b) cell viability, (c-e) invasion and migration of different groups were analyzed by CCK-8, Transwell and wound-healing assays, respectively. (f)The protein expression level of Bcl-2, Bax and cleaved caspase3 was determined by Western blot assay. **p < 0.01, and ***p < 0.001 vs control; ^###^p < 0.001 vs vector.

To explore the regulatory mechanism of SNAI2 in ovarian cancer, SKOV3 cells were transfected with shRNA-SNAI2-1 or shRNA-SNAI2-2 to inhibit the expression level of SNAI2 (Figure 3a). Due to a lower SNAI2 expression in shRNA-SNAI2-1 group than that in shRNA-SNAI2-2 group, shRNA-SNAI2-1 was used for the subsequent experiments. Then, SKOV3 cells were transfected with shRNA-SNAI2 or treated with erastin (30 μM) [23], an inducer of ferroptosis. The results from CCK-8 assay showed that SNAI2 knockdown hugely decreased cell viability of SKOV3 cells, similar to the effect of erastin on cell viability (Figure 3b). In addition, SNAI2 knockdown significantly hindered cell migration and invasion abilities, but promoted cell apoptosis exhibited as the upregulated protein expression of Bax and cleaved caspase3 and the downregulated protein expression of Bcl-2 after shRNA-SNAI2 transfection. Of note, erastin treatment also exerted inhibitory effects on cell migration and invasion abilities and promotive effect on cell apoptosis of SKOV3 cells (Figure 3c-f).

**Figure 4.**
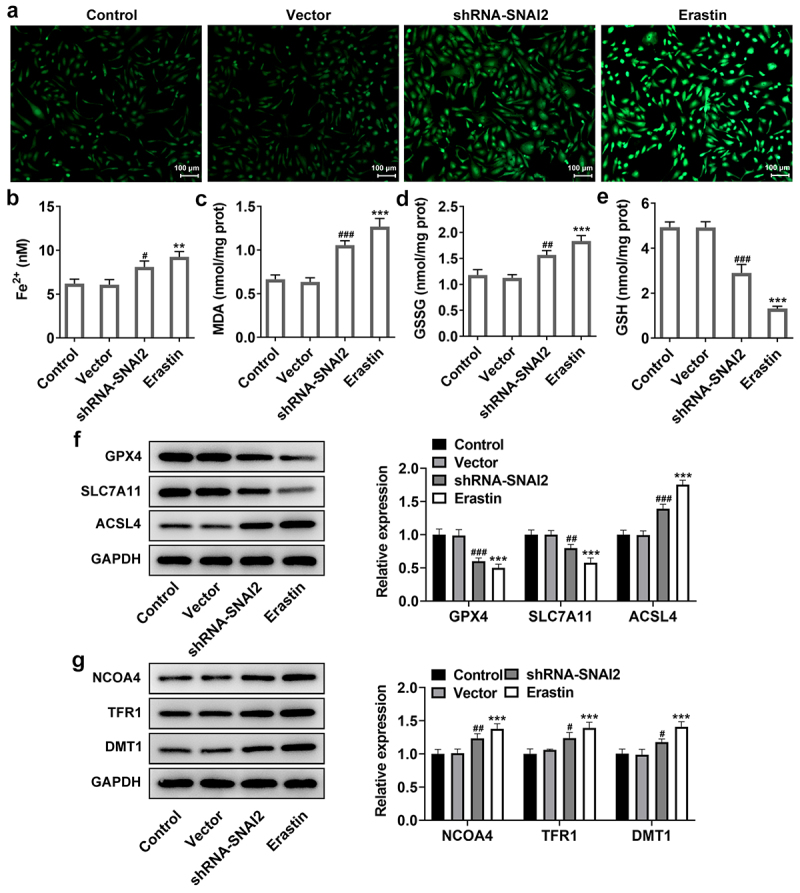
The effect of SNAI2 knockdown on ferroptosis in SKOV3 cells. SKOV3 cells were transfected with shRNA-SNAI2 or vector, or received erastin treatment. The content of (a-b) Fe^2+^, (c) MDA, (d) GSSG and (e) GSH were detected using their corresponding commercial kits. (f) The protein expression of GPX4, SLC7A11, and ACSL4 was measured using Western blot. (g) The protein expression of NCOA4, TFR1, and DMT1 was measured using Western blot. **p < 0.01, and ***p < 0.001 vs control; ^#^p < 0.05, ^##^p < 0.01, and ^###^p < 0.001 vs vector.

Furthermore, SNAI2 knockdown elevated the levels of MDA and GSSG, reduced the level of GSH and Fe^2+^ (Figure 4a-e) and protein expression of lipid peroxide scavengers (GPX4 and SLC7A11), as well as elevating the protein expression of ACSL4 that was responsible for ferroptosis sensitivity (Figure 4f). What’s more, ferroptosis-related proteins, including nuclear receptor coactivator 4 (NCOA4, a selective cargo receptor for the selective autophagic turnover of ferritin), divalent metal transporter 1 (DMT1) and transferrin receptor 1 protein (TFR1), were detected. The results showed that SNAI2 knockdown aggrandized the expression level of NCOA4, DMT1 and TFR1 (Figure 4g). Meanwhile, the effects of erastin on ferroptosis in SKOV3 cells kept the similar trend with SNAI2 knockdown (Figure 4a-g). Taken together, the results suggested that SNAI2 knockdown could promote ferroptosis in SKOV3 cells, likely accounting for its inhibitory effect on cell migration and invasion abilities in ovarian cancer.

### SNAI2 directly bound to SCL7A11 promoter

**Figure 5.**
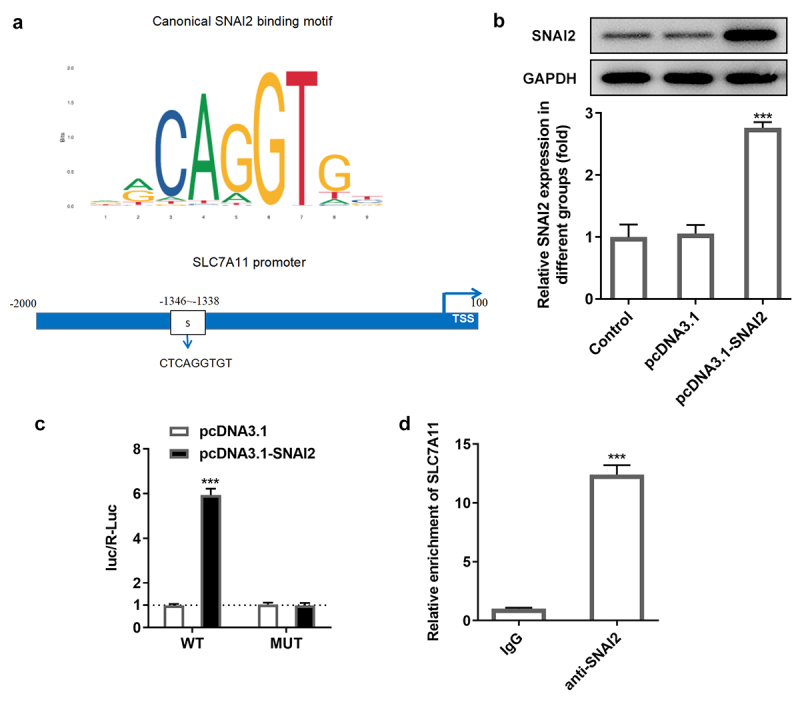
The relationship between SNAI2 and SLC7A11. (a) The potential binding site between SNAI2 and SLC7A11 promoter was predicted by JASPAR bioinformatics website (http://jaspar.genereg.net/). (b) SKOV3 cells were transfected with pcDNA3.1 or pcDNA3.1-SNAI2, and the protein expression level of SNAI2 was measured by Western blot. (c) SKOV3 cells were co-transfected with SLC7A11-WT/SLC7A11-MUT and pcDNA3.1 or pcDNA3.1-SNAI2 using Lipofectamine 3000, and the luciferase activity was examined using dual-luciferase reporter assay kit. ***p < 0.001 vs pcDNA3.1. (d) Chromatin immunoprecipitation (ChIP) assay was performed and the precipitated DNA was detected by qRT-PCR. ***P < 0.001 vs IgG.

To find out the potential mechanism underlying SNAI2 in ovarian cancer, via JASPAR bioinformatics website (http://jaspar.genereg.net/), a potential binding site between SNAI2 and SCL7A11 promoter was identified (Figure 5a). To confirm this connection, SKOV3 cells were transfected with pcDNA3.1 or pcDNA3.1-SNAI2 before luciferase report assay was performed. Western blot assay showed that the protein expression of SNAI2 was significantly increased in pcDNA3.1-SNAI2 group, compared to pcDNA3.1 group, indicating a successful overexpression transfection (Figure 5b). Subsequently, the direct-binding relationship between SNAI2 and SCL7A11 was verified by luciferase report assay and ChIP assay (Figure 5c-d).

### *SNAI2-mediated ferroptosis induction suppressed tumor growth of ovarian cancer* in vivo

**Figure 6.**
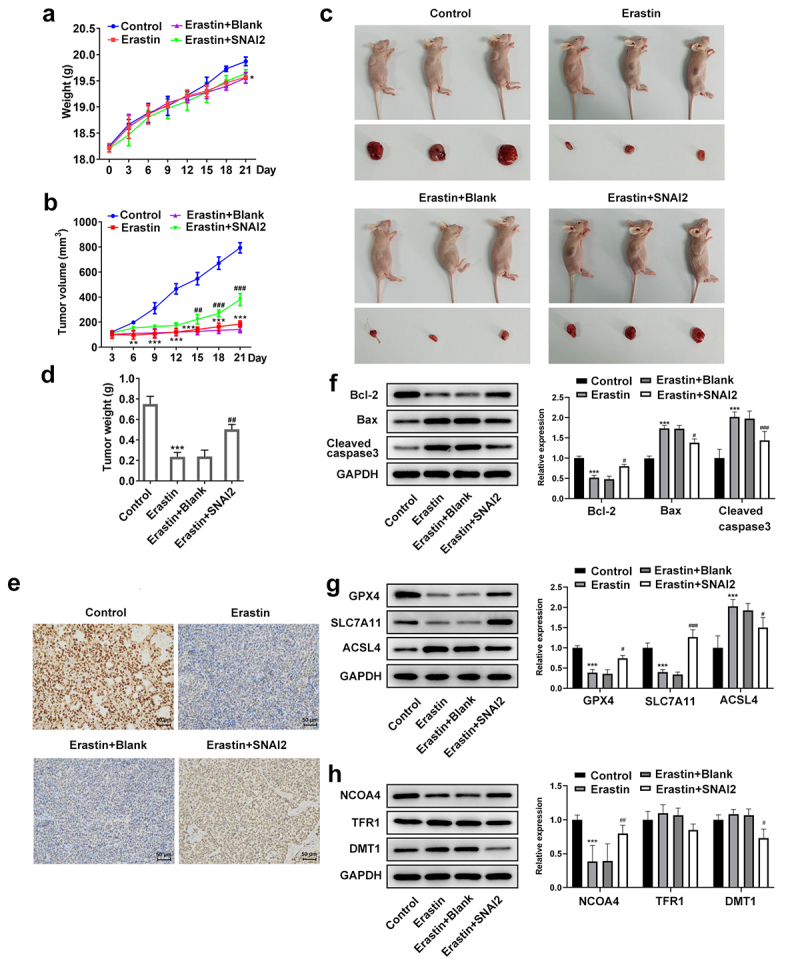
The effect of erastin and SNAI2 overexpression on tumor growth of ovarian cancer *in vivo*. Mice were subcutaneously injected with untransfected or transfected SKOV3 cells to induce tumor formation. Erastin (30 mg/kg intraperitoneally) was applied for treatment. Before sacrifice, the (a) mice weight and (b) tumor volume were monitored and recorded every 3 days. (c) After sacrifice, the tumors were harvested and the tumor size was observed. (d) The tumor weight was also recorded. (e) Immunohistochemical (IHC) assay was conducted to detect the expression of SLC7A11 of tumor tissue. (f) The protein expression of Bcl-2, Bax, and cleaved caspase3 was determined using Western blot. (g) The protein expression of GPX4, SLC7A11, and ACSL4 was determined using Western blot. (h) The protein expression of NCOA4, TFR1, and DMT1 was determined using Western blot. **p < 0.01, and ***p < 0.001 vs control; ^#^p < 0.05, ^##^p < 0.01, and ^###^p < 0.001 vs Erastin+pcDNA3.1.

To further explore the regulatory role of SNAI2-mediated ferroptosis *in vivo*, mice were subcutaneously injected with untransfected or transfected SKOV3 cells to induce tumor formation. As shown in Figure 6a-b, there was no significant difference of mice weight among different groups, while the tumor volume in erastin treatment group was much smaller than that in control group, which was partly restored by SNAI2 overexpression. In addition, after sacrifice, the tumors were harvested and it was observed that the erastin treatment remarkably reduced the tumor size and tumor weight, which was also partly restored when mice were received injection of SKOV3 cells transfected with lentiviral vector of SNAI2 overexpression and received erastin treatment (Figure 6c-d). The IHC assay exhibited that erastin treatment reduced the expression of SCL7A11, in addition to the upregulated SCL7A11 upon injection with SKOV3 cells stably overexpressing SNAI2 (Figure 6e). Moreover, erastin treatment reduced the protein expression of Bcl-2, and elevated the protein expression of Bax and cleaved caspase3, which were then partly abolished by SNAI2 overexpression (Figure 6f). Furthermore, erastin treatment restricted the protein expression of GPX4, SLC7A11, and NCOA4, and elevated the protein expression of ACSL4, indicating a successful induction of ferroptosis, whereas these changes made by erastin were partly abrogated by SNAI2 overexpression. However, the regulatory effect of erastin on TFR1 and DMT1 was not obvious (Figure 6g-h). Taken together, erastin could suppress tumor growth of ovarian cancer, which was partly diminished by SNAI2 overexpression, suggesting that SNAI2 might exert tumor-promoting activity by regulating ferroptosis.

## Discussion

Ovarian cancer is the deadliest of gynecologic malignancies. Even though the great improvements in diagnosis method and high initially response to treatment have achieved in recent years, the five-year survival rate of ovarian cancer is approximately 47% attributed to a delayed diagnosis at an advanced stage and relapse and chemoresistance [3]. Therefore, a better understanding of the molecular mechanism involved in ovarian cancer is critical for developing effective therapeutic strategies.

Ferroptosis, a newly recognized type of regulated cell death driven by iron-dependent phospholipid peroxidation, opens a novel door for the development of ovarian cancer, and is considered as a new therapeutic strategy for ovarian cancer [24]. For example, superparamagnetic iron oxide nanoparticles (SPIONs) were shown to inhibit the proliferation, invasion and drug resistance of human ovarian cancer stem cells by inducing oxidative stress and ferroptosis, thereby reducing ovarian cancer recurrence and metastasis [25]. Emerging evidence implicated that ferroptosis may be an adaptive process which was critical for eradicating the carcinogenic cells [26]. Cancer cells frequently exhibit a high demand for iron to promote cell growth, suggesting that cancer cells are more susceptible to ferroptosis [27,28]. In this study, we firstly verified an occurrence of ferroptosis in ovarian cancer, which was presented as an increased level of iron and an accumulation of lipid peroxide in a series of ovarian cancer cell lines (SKOV3, A2780, and CAOV3), compared to the human normal ovarian cell line IOSE-80. Erastin is a small molecule that targets SLC7A11 to prevent cystine import and cause GSH depletion, thereby inducing ferroptosis [4,29]. It has been reported that erastin is able to enhance the sensitivity of radiotherapy and chemotherapy, and trigger autophagic death, so as to hinder the cancer progression and improve the therapeutic effects of chemoradiotherapy [29–31]. Our findings support the anti-tumor property of erastin in ovarian cancer, evidenced by its inhibitory effects on cell viability, migration, invasion of SKOV3 cells *in vitro*, and on tumor growth *in vivo*, demonstrating that induction of ferroptosis could effectively hinder the development of ovarian cancer.

Given that erastin is a specific inhibitor of SLC7A11, targeting SLC7A11 may be an effective way to mediate ferroptosis, thus participating into tumorigenesis and development. It was reported that lidocaine was exhibited to promote ferroptosis and repress tumor growth of ovarian cancer by regulating miR-382-5p/SLC7A11 axis [32]; PARP inhibition promoted ferroptosis in BRCA mutant ovarian cancer via repressing SLC7A11 [33]. The existing evidence above emphasized the great importance of SLC7A11 in modulating ferroptosis in ovarian cancer, hence a better understanding of the regulatory mechanism of SLC7A11 on the initiation and development of ferroptosis is urgently required to help us develop effective therapeutics in ovarian cancer. To our surprise, the bioinformatics analysis from JASPAR bioinformatics website (http://jaspar.genereg.net/) disclosed a potential binding relationship between SNAI2 and SLC7A11 promoter, which was further verified by luciferase reporter and ChIP assays. The critical role of SNAI2 in regulating cancer occurrence and metastasis, as well as modulating chemotherapy sensitivity, has been uncovered in multiple cancer diseases, including breast cancer, colorectal cancer, and ovarian cancer, via inducing epithelial-mesenchymal transition or other pathways [15,34,35], but whether SNAI2 is involved in or regulates ferroptosis in cancer is poorly understood. The existing data in this study not only demonstrated the anti-tumor effect of SNAI2 knockdown on ovarian cancer, but also illustrated the promotive effect of SNAI2 knockdown on ferroptosis. In addition, SNAI2 knockdown remarkably repressed the protein expression of SLC7A11. The role of SNAI2 knockdown resembled to erastin in ovarian cancer. Considering the binding relationship between SNAI2 and SLC7A11 promoter as aforementioned, it is suggested that SNAI2 knockdown may promote ferroptosis and suppress ovarian cancer progression by directly targeting to and downregulating SLC7A11. Furthermore, the anti-tumor and pro-ferroptosis effects of erastin on ovarian cancer *in vivo* were partially weakened by SNAI2 overexpression, further verifying the critical role of SNAI2-mediated ferroptosis in ovarian cancer progression.

Anyway, we acknowledged some limitations in this study. Firstly, the SLC7A11 promoter and GPX4 promoter were both found to be bound to SNAI2, while we only verified the binding relationship between SLC7A11 and SNAI2. It is still unclear that the regulatory effect of SNAI2 on GPX4 expression is directly determined by their direct-binding relationship or just indirectly impacted by SLC7A11. Moreover, ferroptosis is an emerging and complicated topic in cancer research in recent years, and more research about the regulatory pathways or mechanisms is deserved to be explored.

## Conclusion

In conclusion, we disclosed that SNAI2 knockdown suppressed tumorigenesis and development of ovarian cancer by promoting ferroptosis. Moreover, SNAI2 could directly bind to SLC7A11 promoter and regulate SLC7A11 expression, which might be the potential mechanism underlying SNAI2-regulated ferroptosis in ovarian cancer. Our findings elucidated the molecular mechanism of ferroptosis-mediated ovarian cancer and provided targets for ovarian cancer treatments.

## Data Availability

All data generated or analyzed during this study are included in this published article.
